# Weaning Markedly Affects Transcriptome Profiles and Peyer’s Patch Development in Piglet Ileum

**DOI:** 10.3389/fimmu.2015.00630

**Published:** 2015-12-15

**Authors:** Ryo Inoue, Takamitsu Tsukahara, Masako Nakatani, Mie Okutani, Ryoichiro Nishibayashi, Shohei Ogawa, Tomoko Harayama, Takayuki Nagino, Hironori Hatanaka, Kikuto Fukuta, Gustavo A. Romero-Pérez, Kazunari Ushida, Denise Kelly

**Affiliations:** ^1^Laboratory of Animal Science, Kyoto Prefectural University, Kyoto, Japan; ^2^Gut Immunology Group, Rowett Institute of Nutrition and Health, University of Aberdeen, Aberdeen, UK; ^3^Kyoto Institute of Nutrition and Pathology, Kyoto, Japan; ^4^Technical Center, Toyohashi Feed Mills, Shinshiro, Japan

**Keywords:** neonatal piglets, weaning, ileal mucosa, Peyer’s patches, DNA microarray, metacore pathway analysis, transcriptome

## Abstract

Transcriptome analyses were conducted on the ileal mucosa of 14- to 35-day-old piglets to investigate postnatal gut development during suckling and postweaning. The transcriptome profiles of 14-day-old suckling piglets showed a considerably higher number of differentially expressed genes than did those of 21-, 28-, and 35-day olds, indicating an intensive gut development during the first 14–21 postnatal days. In addition, the analysis of biological pathways indicated that *Chemotaxis Leucocyte chemotaxis* was the most significantly affected pathway in suckling piglets between 14 and 21 days of age. Weaning negatively affected pathways associated with acquired immunity, but positively affected those associated with innate immunity. Interestingly, pathway *Chemotaxis Leucocyte chemotaxis* was found positively affected when comparing 14- and 21-day-old suckling piglets, but negatively affected in 28-day-old piglets weaned at 21 days of age, when compared with 28-day-old suckling piglets. Genes *CXCL13*, *SLA-DOA* (MHC class II), *ICAM1*, *VAV1*, and *VCAM1* were involved in the pathway *Chemotaxis Leukocyte chemotaxis* and they were found to significantly change between 14- and 21-day-old suckling piglets and between groups of suckling and weaned piglets. The expression of these genes significantly declined after weaning at 14, 21, and 28 days of age. This decline indicated that *CXCL13*, *SLA-DOA*, *ICAM1*, *VAV1*, and *VCAM1* may be involved in the development of Peyer’s patches (PP) because lower gene expression clearly corresponded with smaller areas of PP in the ileal mucosa of piglets. Moreover, weaning piglets prior to a period of intensive gut development, i.e., 14 days of age, caused significant adverse effects on the size of PP, which were not reverted even 14 days postweaning.

## Introduction

Development of the gut of growing piglets during early postnatal days is strongly associated with feeding efficiency and susceptibility to pathogens later in life. For example, a pig gut with a well-developed villous/crypt architecture has increased absorptive capacity that results in a higher feed conversion rate (1). In addition, maturation of the porcine mucosal immune system with functional lymphoid organs, including the aggregated lymphoid follicles, provides enhanced protection against pathogens (2, 3). For practical purposes, the aggregated lymphoid follicles are commonly referred to as Peyer’s patches (PP). PP are key components in intestinal immunity and, in human, as much as 46% of PP can be found in the distal 25 cm of ileum (4). Similar PP functions and distribution are likely found in pigs (5, 6). Hence, to maintain the health of growing pigs, it is necessary to ensure a sound development of the digestive–absorptive systems, as well as the mucosal immune structures of the gut such as ileal PP.

The gut of a piglet becomes structurally and functionally comparable with that of an adult at ~2 months of age (7, 8). Nonetheless, intensive development of many parameters associated with the digestive–absorptive and mucosal immune systems is observed during early postnatal life, as piglets still suckle. In the case of the digestive–absorptive system, at ~3 weeks of age lactase activity in the small intestine decreases in parallel with an increase in activity of maltase and sucrase (1, 9). With respect to the mucosal immune system, notable increases in the number of lymphocytes and enlargement of PP (10, 11), as well as marked structural development of the villous architecture (12) have been observed in the small intestinal *lamina propria* during the first 3 weeks of life.

Weaning, particularly early weaning, has a considerable impact on porcine gut development. For instance, weaning significantly increases maltase activity in the small intestine, while simultaneously decreasing lactase activity (13). Furthermore, after weaning, the variety and number of antigens stimulating the mucosal immune system of piglets increase, which contributes to bolster the immune level in the gut, e.g., deployment of more T cells and macrophages/granulocytes to the villous *lamina propria* (14). Moreover, decreases in villous height and increases in crypts depth have been reported by several studies, which clearly exemplify the marked effect of weaning on gut development (15–18).

Transcriptome analysis has been shown to be a useful tool for elucidating gene expression in intestinal tissues of pigs (19). For example, microarray analysis-based bioinformatics have identified in jejunal tissues of weaned piglets: (a) several biological pathways associated with the immune response such as *Natural killer cell mediated cytotoxicity* and *Chemokine signaling pathway* (20) and (b) immune response-related genes affected by foods components such as carbohydrates and fatty acids (21) and amino acids (22). Moreover, recent bioinformatics work has investigated differential gene expression in jejunal (19, 23) and ileal (23, 24) PP. These authors found differential transcriptomic profiles in intestinal tissues, as nutritional pathways were enriched in jejunal PP, whereas immune pathways were overexpressed in ileal PP. Nonetheless, little is known about the identity of biological pathways involved in the development of ileal PP and how enrichment of these pathways and expression of genes related to them affect the development of gut of healthy weaned piglets.

In the present study, first, we analyzed changes in the transcriptome of the ileal mucosa of suckling piglets during early postnatal life, to enhance our understanding of pig’s gut development. Second, we compared the transcriptome of the ileal mucosa of suckling piglets and piglets weaned 21 days after birth, but of same age at the time of comparison, to elucidate the effect of weaning on the developing gut. Third, we assessed the effect of weaning on immune parameters at various ages (14, 21, and 28 days old), with emphasis on biological pathways *Chemotaxis Leukocyte chemotaxis* and *Immune response IFN alpha/beta signaling*, because data suggested that these two pathways were significantly affected by weaning. Last, we evaluated the effect of weaning on ileal PP development, due to pathway *Chemotaxis Leukocyte chemotaxis* was seemingly involved in the development of ileal PP.

## Materials and Methods

### Animals

All piglets were crossbred (Landrace × Large white × Duroc) and bred at the Technical Center of the Toyohashi Feed Mills (Shinshiro, Aichi, Japan). For this work, piglets were obtained from different herds due to the number of animals available was limited. Thus, 30 and 12 piglets were obtained from litters of four and three different sows, respectively. Based on body conditions at the time of group allocation, all selected piglets were tentatively considered suitable for tissue sampling. The average body weight of piglets at farrowing is shown in Table 1. Piglets received a typical commercial diet for weanlings (JustOne Sprout; Toyohashi Feed Mills, Aichi, Japan). This study was carried out in accordance with guidelines for animal studies issued by the Experimental Animal Committee of Kyoto Prefectural University and its protocol approved by this Committee (Approval number: KPU240410).

**Table 1:** **Average body weight of piglets at farrowing and slaughter at different time points**.

Piglet group	Average body weight (kg) at farrowing (*n* = 4)	Average body weight (kg) at slaughter (*n* = 4)
**Suckling**		
S1D	1.990 ± 0.03	1.990 ± 0.03
S7D	1.600 ± 0.06	3.650 ± 0.16
S14D	1.370 ± 0.02	5.250 ± 0.17
S21D	1.300 ± 0.01	6.950 ± 0.20
S28D	1.490 ± 0.06	10.440 ± 0.27
S35D^a^	1.590 ± 0.02	14.600 ± 0.17
21W28D	1.590 ± 0.07	9.090 ± 0.27
21W35D^a^	1.670 ± 0.04	12.030 ± 0.17
**Weaned**		
14W21D	1.700 ± 0.04	5.950 ± 0.17
14W28D	1.890 ± 0.03	8.900 ± 0.09
28W35D	1.620 ± 0.07	12.430 ± 0.35

*Twenty-three piglets from the suckling group were killed and their ileal tissues sampled at 1 (S1D), 7 (S7D), 14 (S14D), 21 (S21D), 28 (S28D), and 35 (S35D) days of age. Four piglets from the suckling group were weaned and killed, and their ileal tissues sampled at 21 and 28 days of age, respectively (21W28D). Three piglets from the suckling group were weaned, and killed, and their ileal tissues sampled at 21 and 35 days of age, respectively (21W35D)*.

*^a^The number of S35D and 21W35D piglets was three each. Eight piglets were weaned at 14 days of age; four were killed, and their ileal tissues sampled at 21 days of age (14W21D), and the other four at 28 days of age (14W28D). Four piglets were weaned at 28 days of age and killed, and their ileal tissues sampled at 35 days of age (28W35D)*.

### Differences in Ileal Transcriptome Between Suckling at Various Ages and Weaning

From a pool of 30 piglets, seven piglets were randomly separated and weaned at 21 days of age (weaned piglet group). Four and three weaned piglets were killed and their ileal tissues sampled 7 (21W28D) and 14 (21W35D) days postweaning, respectively. The remaining piglets stayed with their sows as intensive suckling piglets (sucking piglet group). To ensure that only maternal milk was consumed by the piglets, they were kept from accessing the sow’s diet. Four suckling piglets were killed and their ileal tissues sampled at 1 (S1D), 7 (S7D), 14 (S14D), 21 (S21D), and 28 (S28D) days of age, and the remaining three at 35 days of age (S35D). Only those piglets with acceptable body conditions according to age at slaughter time were finally chosen for sampling. The average body weight of piglets slaughtered at different time points is shown in Table 1. Ileal mucosal samples of all piglets except for those of S1D and S7D were used for microarray analysis.

### Effect of Weaning and Postweaning at Different Ages

From a pool of 12 piglets, eight, and four piglets were separated from sows and weaned at 14 and 28 days of age, respectively. Four piglets weaned at 14 days of age were killed and their tissues sampled at 21 days of age (14W21D) and the remaining four at 28 days of age (14W28D). The piglets weaned at 28 days of age were killed and their tissues sampled at 35 days of age (28W35D). The average body weight of piglets slaughtered at different time points is shown in Table 1. Only those piglets with acceptable body conditions according to age at slaughter time were finally chosen for sampling. Total RNA from the ileal mucosal samples of all piglets was used for real-time PCR and PP histological analyses.

### Tissue Sampling

Piglets were killed by exsanguination after anesthetizing them with an intraperitoneal injection of sodium pentobarbital (Somnopentyl; Kyoritsu, Tokyo, Japan). To remove the entire gut, a midline abdominal incision was cut through the skin; subsequently, the small intestine was excised from the gut. A portion consisting of 15% of the total length, measured from the ileoceacal valve, was collected and regarded as ileum (25, 26). The proximal and distal sections (2 cm long) of this segment were used for histological and gene expression analyses, respectively. Sections were slit lengthwise and washed with saline solution to remove the contents. The proximal section was fixed using a 10% phosphate-buffered formaldehyde solution (v/v). Next, the membrane overlying the ileum was gently scraped from the distal portion with a clean slide glass and immersed into RNAlater (Sigma, Tokyo, Japan). This tissue was regarded as the ileal mucosa, as previously reported (27, 28). Samples were kept at 4°C overnight and subsequently stored at −80°C until used for gene expression analysis.

### Microarray Hybridization

Total RNA was extracted from ileal mucosal samples collected from S14D, S21D, S28D, S35D, 21W28D, and 21W35D piglets. Total RNA extraction and evaluation of RNA integrity were as described by Mulder et al. (29). Only RNA samples showing RNA integrity numbers (RIN) >8.0 were used for the microarray analyses. Biotin-labeled cRNA was synthesized from 100 ng of total RNA using the GeneChip 3′IVT Express kit (Affymetrix, Santa Clara, CA, USA), as per the manufacturer’s instructions. The quality of cRNA was determined using an Agilent 2100 Bioanalyzer (Agilent Technologies, Workingham, UK). Hybridization with the GeneChip Porcine Genome Array (Affymetrix), chip scanning, and image quality analysis were conducted as described elsewhere (29).

### Real-Time PCR Analysis of Differentially Expressed Genes

The expression levels of genes selected on the basis of microarray analyses were further evaluated by real-time PCR. Total RNA was extracted from all samples as mentioned above, and complementary DNA was synthesized from each total RNA sample (500 ng), including those used for microarray analyses, using a RevaTra Ace qPCR RT kit (TOYOBO, Osaka, Japan), as per the manufacturer’s instructions. Real-time PCR was conducted using a LightCycler 480 instrument (Roche Applied Science, Tokyo, Japan). Specific primers and probe sets for each gene were designed with the online Universal ProbeLibrary Assay Design Center (Roche Applied Science). Sequences of the primers and probes used in the current study are shown in Table S1 in Supplementary Material. β-actin was used as the reference gene, and PCR conditions and analyses conducted for all genes were as described elsewhere (30).

### Measurement of the Area of Ileal Peyer’s Patches

All fixed tissue samples were used. Tissues were further cross-sectioned with an approximate length of 10 mm. Sections were individually embedded in paraffin wax. Microsections of 4 μm thickness were prepared and stained with hematoxylin and eosin (H&E) for light microscopic examination. Individual areas of lymphoid follicles in all visible PP were measured using a light microscope (BX51, Olympus, Tokyo, Japan) equipped with a digital camera (DP25, Olympus) and image analysis software (DP2-BSW, Olympus). The PP area value for each tissue sample was calculated by adding the individual values of follicular areas in a cross-microsection. A follicular area in PP was determined based on a definition previously reported (10).

### Data Analysis

To minimize technical variation between array chips, microarray data were normalized using the GeneChip Robust Multiarray Average (gcRMA) method and log-transformed with freely available R^1^ and Bioconductor^2^ software packages. Converted microarray data were statistically analyzed using CLC Main workbench software version 7.6.4 (Qiagen, Tokyo, Japan). Age related differences in the expression value of each gene probe in the suckling and weaned piglet groups were analyzed by one-way ANOVA. Differences between consecutive age groups of suckling piglets (S14D vs. S21D, S21D vs. S28D, and S28D vs. S35D) and between suckling and weaned piglet groups of same age were determined by Student’s *t*-test. A basic cut-off *P* < 0.05, and −2 ≤ fold change ≥ 2 was used to determine the differentially expressed genes. This was in concordance with a study of the Microarray Quality Control (MAQC) (31), which recommends the use of fold change ranking plus a non-stringent *P* cut-off as a baseline practice to generate more reproducible differentially expressed genes. However, for MetaCore analysis, false discovery rate was considered and thus, *q*-value (32) was calculated using the Bioconductor package *qvalue*. Genes at *P* < 0.05, −2 ≤ fold change ≥ 2 and having a *q*-value < 0.1 were imported into MetaCore analytical software (GeneGo, St. Joseph, MI, USA) to generate pathway maps, as carried out by Mulder et al. (29). Integrated pathway enrichment analysis was conducted using the knowledge-based canonical pathways and endogenous metabolic pathways.

Delta Ct value (ΔCt) was used for the statistical analysis of gene expression evaluated by real-time PCR. Differences between consecutive age groups of suckling piglets and between suckling and weaned piglet groups of same age were analyzed using the Student’s *t*-test. A *P*-value < 0.05 was considered statistically significant.

Differences in PP area between consecutive age groups of suckling piglets and between suckling and weaned piglet groups of same age were analyzed by Student’s *t*-test. A *P* value < 0.05 was considered statistically significant.

## Results

### Microarray

Raw and processed microarray data were submitted to the Gene Expression Omnibus (accession number GSE65008,^3^ available from October/01/2015).

### Gene Expression at Different Ages in Suckling Piglets

**Figure 1:**
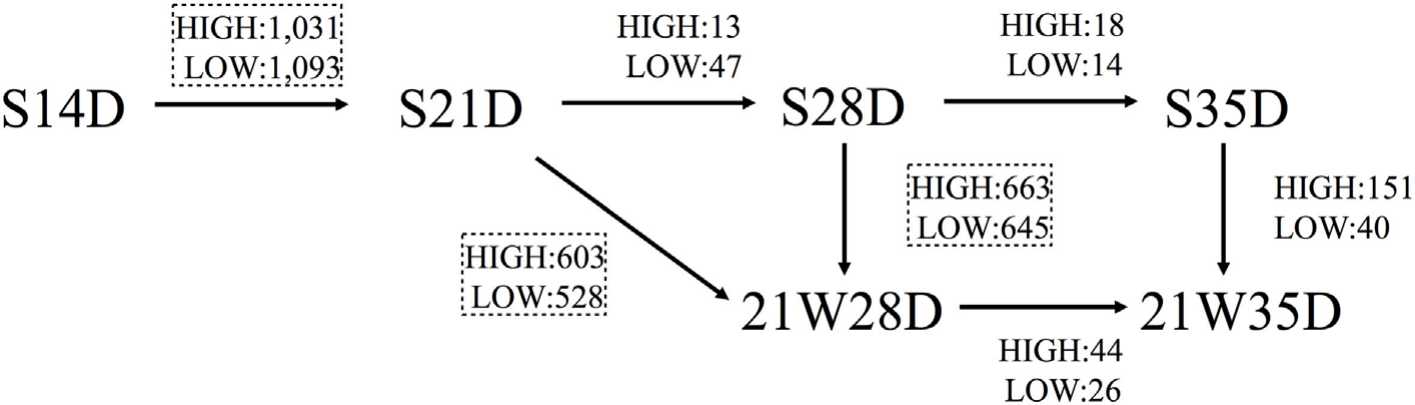
**Differentially expressed genes in piglets**. Figure shows the number of genes differentially expressed (*P* < 0.05, −2 ≤ fold change ≥ 2) in suckling piglets killed and tissue sampled at 14 (S14D), 21 (S21D), 28 (S28D), and 35 (S35D) days of age, and piglets weaned at 21 days of age and killed and tissue sampled 7 (21W28D) and 14 (21W35D) days postweaning. Student’s *t*-test was carried out on the normalized and transformed microarray data and the number of significantly affected genes was calculated. HIGH and LOW denote the number of genes expressed significantly higher and lower, respectively, in piglets at the arrowhead (e.g., S21D) compared with piglets at the other end of the arrow (e.g., S14D). Comparisons between piglets identifying more than 1,000 differentially expressed genes are enclosed in dashed squares. Pathway analysis of the genes in the dashed squares is shown in Table 2.

**Figure 2:**
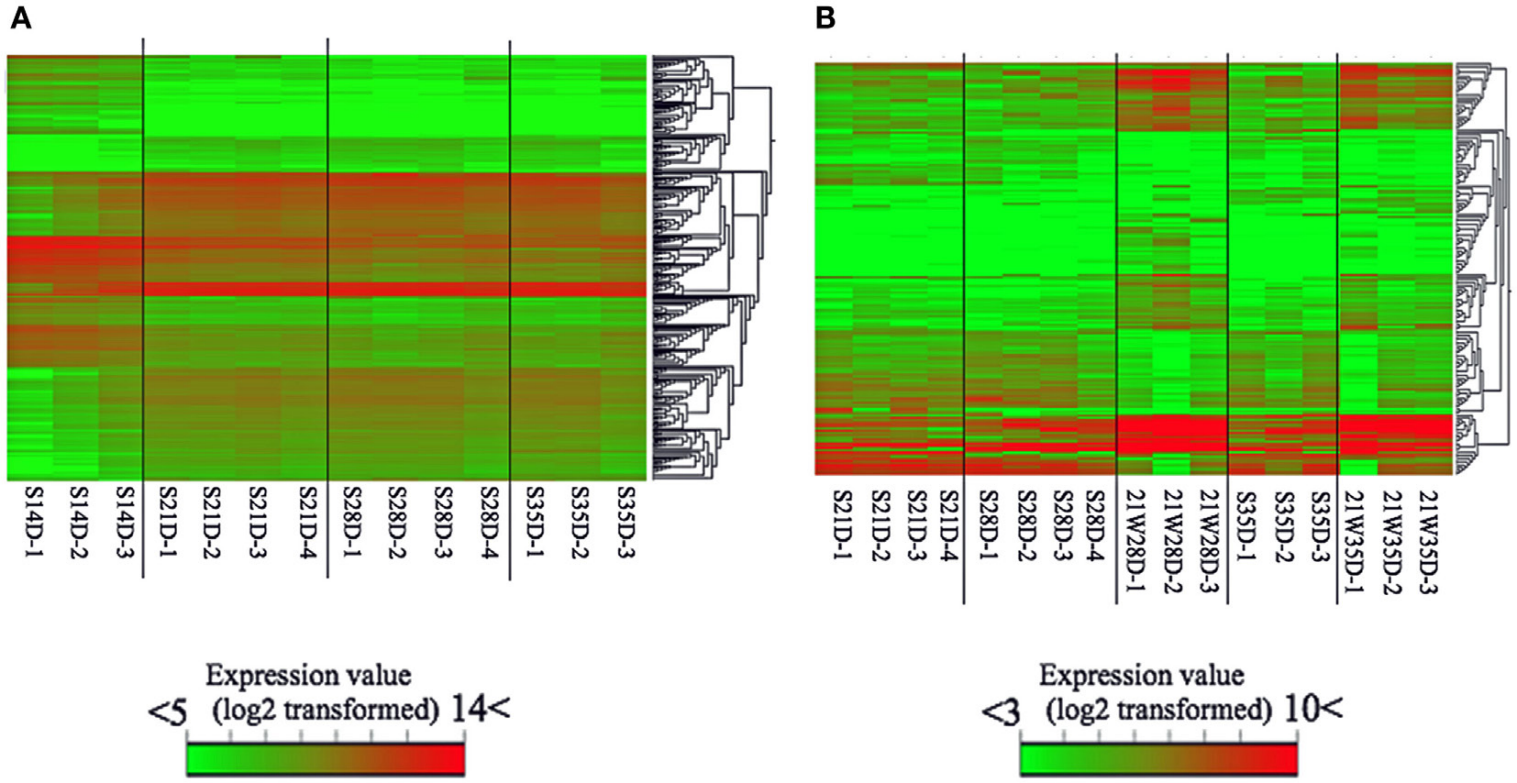
**Microarray-based heat map of differentially expressed gene probes in suckling (A) and weaned (B) piglets of same age**. The heat map was produced by hierarchical clustering of the gene probe set data that met the following criteria: **(A)** ANOVA; *P* < 0.05, −4 ≤ fold change ≥ 4 and **(B)** ANOVA; *P* < 0.05, −3 ≤ fold change ≥ 3. The data of weaning age (S21D) was included to generate data from weaned and suckling piglets of same age.

The number of genes differentially expressed (*t*-test; *P* < 0.05) between S14D and S21D piglets was 2,124, half of which (high: 1,031; low: 1,093) were significantly higher in S21D piglets (Figure 1). In contrast, the number of genes differentially expressed between S21D and S28D and S28D and S35D piglets was only 60 (high: 13; low: 47) and 32 (high: 18; low: 14), respectively (Figure 1). The heat map shown in Figure 2 was generated based on differentially expressed gene probes in ileal tissues of suckling piglets (ANOVA; *P* < 0.05, −4 ≤ fold change ≥ 4). The heat map clearly shows that transcriptome profiles drastically changed from S14D to S21D piglets and older (Figure 2A).

### Effect of Weaning on Gene Expression

Weaning piglets at 21 days of age markedly affected gene expression in ileal tissues. For example, the number of genes differentially expressed between S21D and 21W28D piglets was 1,131 (high: 603; low: 528), that is, more than 18 times than that between S21D and S28D piglets, and the number of genes differentially expressed between S28D and 21W28D was 1,308 (high: 663; low: 645), half of which (645 genes) showed a significant low expression at 21W28D (Figure 1). The heat map clearly shows that the transcriptome profiles between S28D and 21W28D piglets were different (Figure 2B). In addition, the number of genes differentially expressed between S35D and 21W35D piglets was almost sevenfold smaller than those expressed in 28-day old piglets (Figure 1).

### Pathways Identified in Suckling Piglets

The top 10 biological pathways that significantly exerted effects on suckling piglets between 14 and 21 days of age are listed in Table 2. The most significantly enriched pathway based on the genes highly expressed in suckling piglets at 21 days of age (positively affected pathway) was *Chemotaxis Leucocyte chemotaxis*, with 13 genes from a total of 75 analyzed. Two pathways related to immunity were also positively between 14 and 21 days of age. Most enriched pathways corresponding to genes that had a significantly lower expression at 21 days of age (negatively affected pathway) were related to cystic fibrosis transmembrane conductance (CFTR) regulation and cell adhesion (Table 2). Although all differentially expressed genes (*P* < 0.05, −2 ≤ fold change ≥ 2) between 21 and 28 days of age, and between 28 and 35 days of age showed a *q*-value of more than 0.1, pathway analyses were performed using these gene sets for reference purposes. The results can be found in Table S2 in Supplementary Material.

**Table 2:** **MetaCore pathway analysis of differentially expressed genes between ileal mucosal tissues of related piglet groups**.

	*P* value	Significance^a^	Total of genes^b^
**S14D vs. S21D (positively affected from S14D to S21D)**			
Chemotaxis leukocyte chemotaxis	1.570E−07	13	75
DNA damage/ATM-ATR regulation of G2/M checkpoint	3.903E−07	8	26
G-protein signaling/regulation of RAC1 activity	5.164E−07	9	36
Immune response/immunological synapse formation	6.746E−07	11	59
DNA damage/mismatch repair	7.942E−07	7	20
Immune response/CCR3 signaling in eosinophils	1.564E−06	12	77
Cell cycle/role of APC in cell cycle regulation	2.266E−06	8	32
DNA damage/role of Brca1 and Brca2 in DNA repair	1.634E−05	7	30
Cell cycle/role of 14-3-3 proteins in cell cycle regulation	2.571E−05	6	22
DNA damage/ATM-ATR regulation of G1/S checkpoint	2.573E−05	7	32
**S14D vs. S21D (negatively affected from S14D to S21D)**			
wtCFTR and delta508 traffic/clathrin-coated vesicles formation (norm and CF)	1.673E−04	5	19
Cardiac hypertrophy/NF-AT signaling in cardiac hypertrophy	5.545E−04	8	65
Cell adhesion/gap junctions	1.578E−03	5	30
Transcription/ChREBP regulation pathway	2.842E−03	4	21
Cell adhesion/tight junctions	3.623E−03	5	36
Transport/clathrin-coated vesicle cycle	4.437E−03	7	71
Regulation of CFTR activity (norm and CF)	6.546E−03	6	58
Cell adhesion/chemokines and adhesion	8.566E−03	8	100
CFTR-dependent regulation of ion channels in airway epithelium (norm and CF)	8.647E−03	5	44
Development/regulation of epithelial-to-mesenchymal transition (EMT)	1.050E−02	6	64
**S21D vs. 21W28D (positively affected from S21D to 21W28D)**			
Immune response/alternative complement pathway	1.156E−07	9	39
Regulation of metabolism/bile acids regulation of glucose and lipid metabolism via FXR	1.280E−05	7	37
Role of ZNF202 in regulation of expression of genes involved in atherosclerosis	7.329E−05	5	21
Immune response/lectin induced complement pathway	8.556E−05	7	49
Triacylglycerol metabolism p.1	2.825E−04	7	59
Immune response/antigen presentation by MHC class I	3.125E−04	5	28
Glycine, serine, cysteine, and threonine metabolism	3.532E−04	10	123
Glycine, serine, cysteine, and threonine metabolism/rodent version	4.021E−04	10	125
Apoptosis and survival/granzyme B signaling	5.965E−04	5	32
Cholesterol biosynthesis	6.591E−04	8	88
**S21D vs. 21W28D (negatively affected from S21D to 21W28D)**			
Immune response/MIF-mediated glucocorticoid regulation	2.165E−08	7	22
G-protein signaling/regulation of RAC1 activity	1.427E−05	6	36
Immune response/antigen presentation by MHC class II	2.247E−05	4	12
Immune response/MIF-induced cell adhesion, migration, and angiogenesis	6.062E−05	6	46
Immune response/HMGB1/RAGE signaling pathway	1.363E−04	6	53
Immune response/HSP60 and HSP70/TLR signaling pathway	1.514E−04	6	54
Immune response/BCR pathway	1.514E−04	6	54
Immune response/PIP3 signaling in B lymphocytes	3.969E−04	5	42
Development/VEGF signaling and activation	4.437E−04	5	43
Immune response/histamine H1 receptor signaling in immune response	7.431E−04	5	48
**S28D vs. 21W28D (positively affected from S28D to 21W28D)**			
CFTR-dependent regulation of ion channels in airway epithelium (norm and CF)	4.501E−05	7	44
Apoptosis and survival_Caspase cascade	7.274E−04	5	33
Cell adhesion/tight junctions	1.095E−03	5	36
Regulation of metabolism/bile acids regulation of glucose and lipid metabolism via FXR	1.244E−03	5	37
Regulation of CFTR activity (norm and CF)	1.703E−03	6	58
Immune response/IFN alpha/beta signaling pathway	1.756E−03	4	24
Proteolysis/role of Parkin in the ubiquitin-proteasomal pathway	1.756E−03	4	24
Triacylglycerol metabolism p.1	2.031E−03	6	60
Plasmalogen biosynthesis	2.827E−03	6	64
Cell adhesion/gap junctions	4.084E−03	4	30
**S28D vs. 21W28D (negatively affected from S28D to 21W28D)**			
Immune response/BCR pathway	1.421E−08	10	54
Immune response/PIP3 signaling in B lymphocytes	2.000E−08	9	42
Chemotaxis/leukocyte chemotaxis	3.370E−08	11	75
Chemotaxis/CCR4-induced leukocyte adhesion	7.861E−06	6	30
Immune response/NFAT in immune response	1.833E−05	7	51
Immune response/immunological synapse formation	4.839E−05	7	59
Development/EPO-induced PI3K/AKT pathway and Ca(2+) influx	6.765E−05	6	43
Development/EPO-induced MAPK pathway	8.796E−05	6	45
Immune response/CD16 signaling in NK cells	1.334E−04	7	69
Cell cycle/role of APC in cell cycle regulation	1.618E−04	5	32

*^a^Significance (*P* < 0.05) was calculated by comparing the number of differentially expressed genes (*P* < 0.05, −2 ≤ fold change ≥ 2, *q*-value < 0.1) between related piglet groups in each map*.

*^b^Total number of genes in maps assigned to each pathway. Differentially expressed genes were imported into GeneGo Metacore software. Only the top 10 pathways for each comparison are shown*.

### Effect of Weaning on Identified Pathways

The MetaCore enrichment analysis indicated that many pathways associated with immunity were affected by weaning. Pathways associated with acquired immunity was negatively affected in 21W28D piglets in comparison with S21D or S28D, whereas pathways associated with innate immunity seemed to be positively affected (Table 2). For example, when comparing S21D with 21W28D piglets, pathway *Immune response Antigen presentation by MHC class I* was found positively affected but pathway *Immune response Antigen presentation by MHC class II* was found negatively affected. Pathway *Chemotaxis Leucocyte chemotaxis* was found positively affected when comparing S14D with S21D, but enriched as negatively affected in 21W28D piglets when comparing suckling piglets of same age (S28D). At 28 and 35 days of age, pathway *Immune response IFN alpha/beta signaling* was found enriched as positively affected in weaned piglets when compared with suckling piglets (Table 2; Table S2 in Supplementary Material). As above, pathway analyses based on the differentially expressed genes between 21W28D and 21W35D, and between S35D and 21W35D were carried out for reference purposes, although the *q*-value for these genes was higher than 0.1 (Table S2 in Supplementary Material).

### Real-Time PCR Analysis of Gene Expression in Suckling Piglets

**Figure 3:**
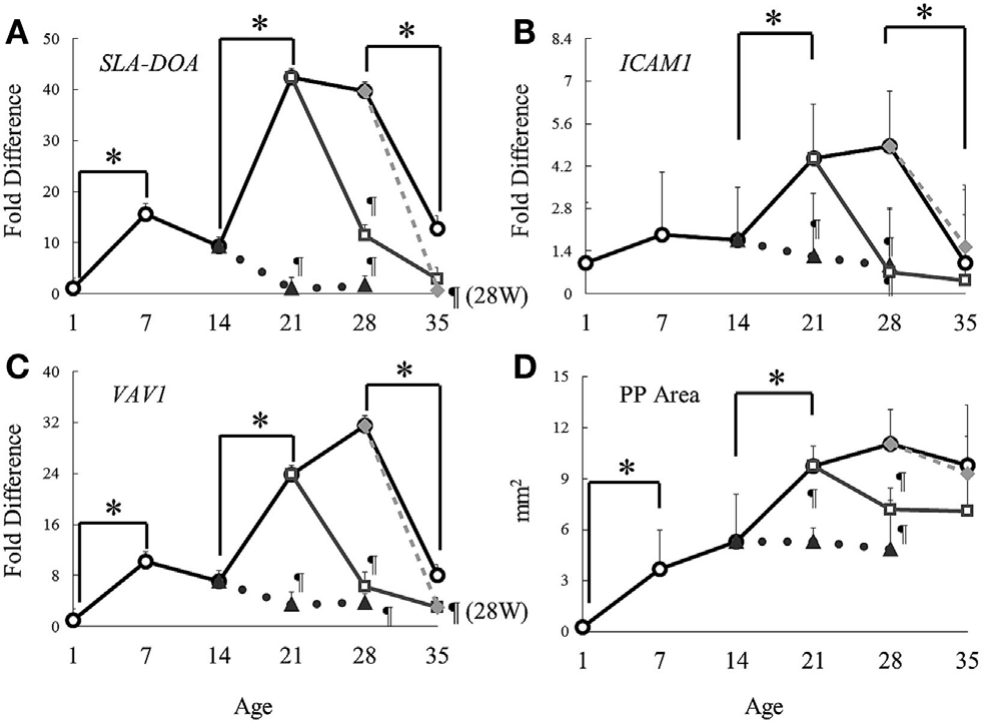
**Dynamics of gene expression and areas of Peyer’s patches during the experimental period**. Symbols: solid line and open circles: suckling piglets; dotted line and solid triangles: piglets weaned at 14 days of age; solid line and open squares: piglets weaned at 21 days of age; gray line and solid rhombuses: piglets weaned at 28 days of age. Gene expression of **(A)***SLA-DOA*, **(B)** *ICAM1*, and **(C)** *VAV1* were analyzed using real-time PCR. The expression of each gene was normalized to that of β-actin. Data are presented as fold differences (mean ± SD) compared with the expression level of S1D. The sum of all visible follicular areas of Peyer’s patches was measured and regarded as **(D)** area of Peyer’s patches. Data are shown as mean ± SD. *P < 0.05 between ages, and ^¶^*P* < 0.05 between weaned and suckling piglets of same age.

Genes *CXCL13*, *SLA-DOA* (MHC class II), *ICAM1*, *VAV1*, and *VCAM1* were selected as involved in the pathway *Chemotaxis Leukocyte chemotaxis* because their expression commonly changed between S14D and S21D and between S28D and 21W28D piglets in the microarray analyses. Real-time PCR analysis showed that the expression of *SLA-DOA* and *VAV1* significantly elevated in suckling piglets from 1 to 7 days of age. Interestingly, from 14 to 21 days of age, all genes in suckling piglets showed a clear elevation of gene expression, of which the level difference was statistically significant (Figure 3; Table 3). Nonetheless, the expression of all genes started to decline markedly in piglets older than 28 days of age (*P* < 0.05 for *SLA-DOA*, *ICAM1*, and *VAV1*; Figures 3A–C).

**Table 3:** **Real-time PCR analyses of selected genes in ileal mucosal tissues of suckling and weaned piglets**.

Gene	Suckling piglet	14W^a^	21W^b^	28W^c^	Age at slaughter and tissue sampling
**Chemotaxis leukocyte chemotaxis pathway**					
*VCAM1*					
	7.01 ± 1.50				1D
	8.12 ± 0.96				7D
	7.83 ± 0.71				14D
	6.26 ± 0.70^§^	9.47 ± 2.00^§^			21D
	6.22 ± 0.38	9.21 ± 1.23^§^	8.03 ± 0.47^§^		28D
	7.42 ± 1.86		8.34 ± 1.36	10.51 ± 1.23^§^	35D
*CXCL13*					
	8.39 ± 2.79				1D
	8.29 ± 2.96				7D
	8.69 ± 1.32				14D
	6.64 ± 1.98	9.75 ± 1.87^§^			21D
	4.98 ± 0.52	10.38 ± 2.32^||^	8.00 ± 0.95^§^		28D
	6.06 ± 2.12		8.24 ± 4.89	10.22 ± 0.58^§^	35D
**Immune response/IFN alpha/beta signaling pathway**					
*ISG15*					
	6.57 ± 0.26	6.17 ± 1.09			21D
	5.27 ± 0.98	6.94 ± 0.45^§^	3.36 ± 0.80^§^		28D
	8.22 ± 0.67		4.69 ± 0.24^§^	5.37 ± 1.00^§^	35D
*ISG54*					
	9.77 ± 0.86	8.77 ± 3.61			21D
	8.98 ± 1.69	8.66 ± 3.61	7.53 ± 1.81		28D
	11.35 ± 0.74		9.49 ± 0.66^§^	7.55 ± 3.51^§^	35D
*IFI6*					
	14.02 ± 0.99	12.65 ± 5.38			21D
	13.36 ± 1.48	12.09 ± 6.02	12.56 ± 1.44		28D
	16.64 ± 0.98		14.71 ± 0.50^§^	10.40 ± 4.67^§^	35D
*USP18*					
	11.16 ± 0.61	11.13 ± 4.23			21D
	10.34 ± 1.02	12.12 ± 5.42^§^	9.60 ± 1.37		28D
	13.67 ± 0.25		12.54 ± 0.80^||^	11.28 ± 5.22^||^	35D

*^a^Piglets weaned at 14 days of age*.

*^b^Piglets weaned at 21 days of age*.

*^c^Piglets weaned at 28 days of age. PCR values are expressed as mean ΔCt ± SD*.

*Significance was estimated after comparisons between data of chronologically consecutive piglet groups (e.g., 21D vs. 14D suckling piglets) or after comparison with suckling piglets of same age. ^§^*P* < 0.05; ^||^*P* < 0.1. In the data shown, smaller values actually denote higher expression. The fold difference between groups can be appreciated by raising the difference of ΔCt between groups to the power of 2*.

### Real-Time PCR Analysis of Gene Expression in Weaned Piglets

At day 7 postweaning, the expression of all genes involved in the pathway *Chemotaxis Leukocyte chemotaxis* was significantly lower in all weaned piglets when compared with that in suckling piglets of same age (Figure 3; Table 3). Furthermore, in piglets weaned at 14 days of age, an elevation in gene expression similar to that between S14D and S21D piglets was not observed and hence, gene expression was still significantly lower at 14 days postweaning when compared with that in 28-day-old suckling piglets (14W28D vs. S28D). However, differences in gene expression between weaned and suckling piglets were non-significant at 35 days of age due to the marked decline in gene expression in piglets older than 28 days of age mentioned above. The effect of weaning on the expression of genes *ISG15*, *ISG54*, *IFI6*, and *USP18* involved in the pathway *Immune response IFN alpha/beta signaling* was differentially dependent on the weaning age. For example, the expression of *ISG15* and *USP18* was lower 14 days postweaning in piglets weaned at 14 days of age than in 28 days old suckling piglets (S28D). In contrast, the expression of all genes was markedly higher (*P* < 0.05) in 35 days old piglets that were weaned at 21 and 28 days of age than that of genes in 35-day-old suckling piglets (S35D).

### Measurement of the Area of Ileal Peyer’s Patches

**Figure 4:**
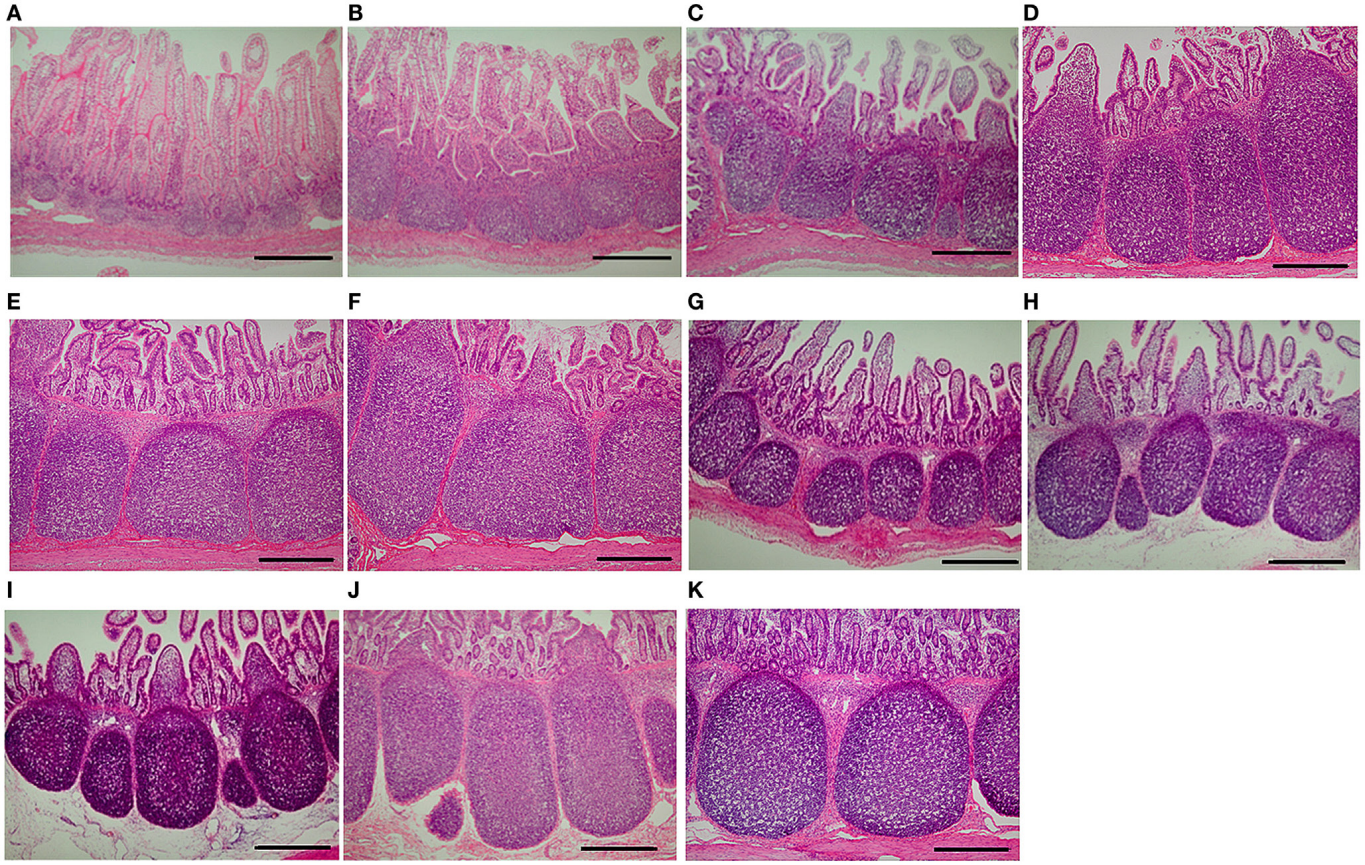
**Peyer’s patches in piglet ileal samples**. Representative images of Peyer’s patches in ileal tissues of suckling piglets killed at **(A)** 1, **(B)** 7, **(C)** 14, **(D)** 21, **(E)** 28, and **(F)** 35 days of age are shown. In addition, the figure also shows representative images of Peyer’s patches in ileal tissues of piglets weaned and killed at **(G)** 14 and 21, **(H)** 14 and 28, **(I)** 21 and 28, **(J)** 21 and 35, and **(K)** 28 and 35 days of age, respectively. Photomicrographs (original magnification ×40; hematoxylin and eosin stain) were taken using a digital camera attached to a light microscope. Bars = 500 μm.

The dynamics of ileal PP areas during the experiment was similar to that of the expression of genes involved in the pathway *Chemotaxis Leukocyte chemotaxis*, particularly with *SLA-DOA* and *VAV1* (Figures 3 and 4). For example, in suckling piglets, ileal PP areas significantly increased from 1 (Figure 4A) to 7 (Figure 4B) and from 14 (Figure 4C) to 21 (Figure 4D) days of age, the latter enlargement being approximately twofold. Except for piglets weaned at 28 days of age (Figure 4K), ileal PP areas were smaller in weaned piglets (Figures 4G–J) than those observed in suckling piglets (Figures 4C,D), being this difference more marked if comparison was made between piglets weaned at 14 days of age (Figure 4C vs. Figures 4G,H), in which PP area size remained essentially unchanged (Figure 3D).

## Discussion

The Affymetrix GeneChip Porcine Genome Array contains 24,123 probe sets and ~20,000 were annotated by (33), of which 11,265 represented unique genes. This powerful tool enabled us to analyze changes in transcriptome profiles and biological pathways in the porcine ileum that occurred during its development or the weaning process. In the present study, the microarray analyses were conducted on the ileal mucosa to comprehensively characterize changes in all cell lineages present in the gut rather than on a specific cell subset (29).

β-actin was the housekeeping gene used in the present work. Like many others, β-actin is expected to be expressed in all cells under normal conditions. However, the use of β-actin as reference gene has been lately questioned as it is argued that constant expression, rather than ubiquity, across tissues should be the decisive attribute when choosing a gene as internal control for experimental expression analysis (34, 35). Nonetheless, gene stability may depend on the type and region of tissues (36), and experiment conditions and species (37). Moreover, recent work (38, 39), including that on gene expression in pig tissues (36, 40), used β-actin as reference for measuring gene expression, with no reported problems. In addition, for further assurance, we evaluated *GAPDH* as an alternative reference gene and found a significant positive correlation (*P* < 0.01) with the expression level of *ACTB*. Based on all this, we considered β-actin a reliable reference gene.

A remarkable change in transcriptome profiles was observed when the analyses of tissues from 14- to 21-day-old suckling piglets were compared. A side-by-side comparison of profiles from S14D and S21D piglets showed that the number of genes differentially expressed was more than 10-fold higher than those found between S21D and S28D or between S28D and S35D piglets. This indicated that a drastic development of ileum took place within a week, between 14 and 21 days of age, but afterward it was only relatively modest. Moreover, MetaCore analyses revealed that in suckling piglets pathways associated with immunity were positively affected in the same period, which also suggests that development of the mucosal immune system also took place within this time.

*Chemotaxis Leukocyte chemotaxis* was the most significantly affected pathway, and putatively involved in immune cell deployment to the ileum. It is of interest that the same pathway and even the same genes were negatively affected by weaning. Real-time PCR analyses demonstrated that weaning significantly attenuated the gene expression involved in this pathway not only at 21 days of age but also at 14 and 28 days of age. In contrast, it was previously reported that a great number of immune cells are deployed to the *lamina propria* of the small intestine due to weaning (41). Therefore, we hypothesized that this pathway was very much involved in the development of PP. Our results confirmed that the dynamics of PP enlargement was very similar to that of genes involved in this pathway. PP areas significantly increased from day 14 to 21 in suckling piglets along with the expression of the genes involved in this pathway, but virtually ceased when the expression of these genes diminished at 35 days of age. In addition, differences in gene expression in tissues from weaned and suckling piglets of same age clearly coincided with size differences in PP areas.

*ICAM1*, *VCAM1*, and *CXCL13* are involved in attraction/retention of hematopoietic cells in developing PP (42) and an increase in *SLA-DOA* may imply activation or proliferation of dendritic cells, as it has been previously suggested that dendritic cells from porcine PP highly expresses MHC class II (43). With regard to Vav-1, the involvement of this GTP exchange factor in the formation/development of PP has not yet been demonstrated. However, previous work reported that Vav-1 was critical for T cell development, and that deficiency in *VAV1* resulted in reduced T cell population in lymph nodes (44). Thus, an increase in this gene may indicate T cell activation or an increase in the number of these cells in developing PP. The reason the low gene expression in 28W35D did not affect PP areas was presumably because gene expression had already peaked before weaning. By contrast, critical maldevelopment of PP observed in tissues of 14W21D piglets, which did not revert even after 14 days postweaning, was possibly due to piglets were weaned before gene expression peaked.

MetaCore analyses also showed that many pathways such as *Immune response BCR pathway* and *Immune response PIP3 signaling in B lymphocytes* associated with immunity, possibly humoral immunity, were negatively affected by weaning. This may be explained by the maldevelopment of PP because they play a critical role in humoral immunity, including production of antibodies (45). Indeed, it is quite possible that underdevelopment of PP observed after weaning resulted in lower expression of chemotactic genes such as *CXCL13*, which in turn caused a reduction of B cell homing and overall immune response (46). In contrast, type 1 IFN signaling pathway (*Immune response IFN alpha/beta signaling pathway*) seemed to be positively affected by weaning. Unexpectedly, real-time PCR analyses showed that the older the piglets were at weaning the larger the effect of weaning on the genes involved in this pathway. Type 1 IFNs have many biological properties (47); thus, the exact role of type 1 IFN signaling on gut development is still not clear. It has been demonstrated that ileum of conventionalized 14-day-old piglets showed activated IFN-γ and type 1 IFN signaling pathways in comparison with germ-free piglets of same age (26). Thus, it is likely that type 1 IFN signaling pathway was activated by the colonization of intestinal microbiota. In contrast, a counteracting inhibitory mechanism must have been activated to maintain intestinal homeostasis as suggested by Chowdhury et al. (26). Because of this inhibitory mechanism, type 1 IFN signaling pathway may be attenuated at an older age as shown by the decreased expression of all evaluated genes associated with this pathway. Among the four genes evaluated (*ISG15*, *ISG54*, *IFI6*, and *USP18*) that were induced by interferon-stimulated gene factor 3 (ISGF3) due to IFN-α stimulation, USP18 is known as a potent negative regulator of type 1 IFN signaling (48), and ISG15 is also suggested as a key negative regulator of the signaling in human by stabilizing USP18 (49). Thus, these two proteins may be involved in this inhibitory mechanism. Furthermore, type 1 IFN signaling pathway has been suggested as affected by the composition of the intestinal microbiota (29). Thus, it can tentatively be theorized that drastic changes in the microbiota composition caused by the lack of maternal milk, i.e., weaning, may have activated this pathway again.

In conclusion, the present study suggested that development of porcine ileum was not gradual but took place stepwise and proceeded intensively during a certain period (< 7 days). Indeed, according to real-time PCR analyses carried out in the present study and the dynamics of PP enlargement, intensive development may have occurred from the age of 1–7 days as well as from the age of 14–21 days. However, the time in which intensive development occurs could slightly change depending on the growing environment as it has a significant effect on the establishment of gut microbiota and overall gut development (29). The results of the present study clearly indicate that weaning before 21 days of age causes a critically adverse effect on gut development of piglets, leading to maldevelopment of PP that did not revert even after 14 days postweaning. The present work is the first to clearly show the effect of age at weaning on the development of PP, based on supporting expression patterns of genes perhaps involved in the process. This finding warrants further evaluation of the relationship between transcriptomic changes during weaning and gut microbiota and/or immune cell population. Future work using a combination of techniques such as laser capture micro dissection and DNA microarray analyses could be highly useful to reveal changes taking place in specific anatomical sites such as PP.

## Conflict of Interest Statement

The authors declare that the research was conducted in the absence of any commercial or financial relationships that could be construed as a potential conflict of interest.

## Footnotes

^1^https://www.r-project.org/

^2^http://bioconductor.org/

^3^https://www.ncbi.nlm.nih.gov/geo/
