# Supplementary material for: Weaning Markedly Affects Transcriptome Profiles and Peyer’s Patch Development in Piglet Ileum
Source: Front Immunol. 2015 Dec 15;6:630. doi: 10.3389/fimmu.2015.00630 (PMC4678207; doi:10.3389/fimmu.2015.00630)
Supplement: Supplementary file 1 [file Table_1.DOCX]

**Supplementary Table 1.** The sequences of primers and probes used in Real-time PCR analysis Listed probe numbers indicate the product number of the Universal ProbeLibrary Set and Human Extension Set sold by Roche Applied Science.

| **Gene** | **Sequences(5'-3')** | **Accession No.** | **Probe number** |
| --- | --- | --- | --- |
| *Chemotaxis Leukocyte chemotaxis pathway* | |  | |
| ***SLA-DOA*(MHC class II)** | F gagccgctctttacgcact | AB012858 | #45 |
|  | R ccagggtctctgtggtgtct |  |  |
| ***VCAM1*** | F ttgacgtgaaaggaagagaaagt | NM_213891 | #88 |
|  | R ccccgatggcaggtattatt |  |  |
| ***ICAM1*** | F ggaaaatgcagccatgaaac | AF156712 | #149 |
|  | R ctgccctggatcaggttc |  |  |
| ***VAV1*** | F ctgtctgttcatctttggtatgct | XM_003480810 | #43 |
|  | R gagcggttggtgaggatg |  |  |
| ***CXCL13*** | F aagcctccggaagagattg | XM_003129101 | #27 |
|  | R cttgtcaggctgaaactcca |  |  |
|  | |  | |
| *Interferon alpha/beta signaling pathway* | |  | |
| ***IFI6*** | F gcatcaggcacagagtaggg | BX676643 | #32 |
|  | R gcgtcttcttttgtctggaaa |  |  |
| ***USP18*** | F ctccttgagacggaggaaga | NM_213826 | #64 |
|  | R agcgaagagctcgtaccg |  |  |
| ***ISG54 (IFIT2)*** | F aatttgttccttgtctgctcct | XM_001928671 | #34 |
|  | R ttcttcatactgaccagcttgc |  |  |
| ***ISG15*** | F agcaacgcctatgaggtctg | NM_001128469 | #112 |
|  | R tgacacacctgctgcttga |  |  |
|  |  |  |  |
| Reference gene |  |  |  |
| ***ACTB*** | F ctaggagcgggttgaggtg | XM_003124280 | #71 |
|  | R ctggtctcaagtcagtgtacaggt |  |  |
