# Supplementary material for: Weaning Markedly Affects Transcriptome Profiles and Peyer’s Patch Development in Piglet Ileum
Source: Front Immunol. 2015 Dec 15;6:630. doi: 10.3389/fimmu.2015.00630 (PMC4678207; doi:10.3389/fimmu.2015.00630)
Supplement: Supplementary file 2 [file Table_2.DOCX]

**Supplementary Table 2.** MetaCore pathway analysis of differentially expressed genes in ileal mucosal tissues of related piglet groups

*Significance (P < 0.05) was calculated by comparing the number of differentially expressed genes between related piglet groups in each map. Differentially expressed genes were determined with a cut-off *P* < 0.05, and -2≤fold change≥2. **Total number of genes in maps assigned to each pathway. Although all differentially expressed genes used for comparisons had a q-value >0.1, they were imported into GeneGo Metacore software to perform pathway analyses only for reference purposes. Only the top ten pathways for each comparison are shown.

|  | ***P* value** | **Significance*** | **Total of genes**** |
| --- | --- | --- | --- |
| **S21D vs S28D (positively affected from S21D to S28D)** |  |  |  |
| wtCFTR and deltaF508 traffic / Membrane expression (norm and CF) | 8.133E-03 | 1 | 34 |
| Immune response / TLR signaling pathways | 1.338E-02 | 1 | 56 |
| Chemotaxis / Leukocyte chemotaxis | 1.790E-02 | 1 | 75 |
| Transport / Intracellular cholesterol transport in norm | 2.146E-02 | 1 | 90 |
|  |  |  |  |
| **S21D vs S28D (negatively affected from S21D to S28D)** |  |  |  |
| Transcription / Role of heterochromatin protein 1 (HP1) family in transcriptional | 5.925E-04 | 2 | 22 |
| silencing |  |  |  |
| Transcription / Role of AP-1 in regulation of cellular metabolism | 1.776E-03 | 2 | 38 |
| Regulation of lipid metabolism / Insulin signaling:generic cascades | 2.707E-03 | 2 | 47 |
| Regulation of lipid metabolism / Insulin regulation of glycogen metabolism | 3.823E-03 | 2 | 56 |
| Immune response / Antigen presentation by MHC class II | 1.996E-02 | 1 | 12 |
| wtCFTR and delta508 traffic / Clathrin coated vesicles formation (norm and CF) | 3.144E-02 | 1 | 19 |
| Development / WNT signaling pathway. Part 1. Degradation of beta-catenin in the | 3.144E-02 | 1 | 19 |
| absence WNT signaling |  |  |  |
| Vitamin K metabolism | 3.469E-02 | 1 | 21 |
| Translation / Regulation of translation initiation | 4.440E-02 | 1 | 27 |
| Cholesterol and Sphingolipids transport / Influx to the early endosome in lung | 4.921E-02 | 1 | 30 |
| (normal and CF) |  |  |  |
|  |  |  |  |
| **S28D vs S35D (positively affected from S28D to S35D)** |  |  |  |
| Immune response / Oncostatin M signaling via JAK-Stat in mouse cells | 6.459E-03 | 1 | 18 |
| Immune response / TLR3 and TLR4 induce TICAM1-specific signaling pathway | 7.174E-03 | 1 | 20 |
| Immune response / Oncostatin M signaling via JAK-Stat in human cells | 7.174E-03 | 1 | 20 |
| Immune response / IFN alpha/beta signaling pathway | 8.605E-03 | 1 | 24 |
| Immune response / IL-27 signaling pathway | 8.605E-03 | 1 | 24 |
| Immune response / Innate immune response to RNA viral infection | 1.003E-02 | 1 | 28 |
| Immune response / IL-6 signaling pathway | 1.039E-02 | 1 | 29 |
| Immune response / Signaling pathway mediated by IL-6 and IL-1 | 1.075E-02 | 1 | 30 |
| Development / CNTF receptor signaling | 1.218E-02 | 1 | 34 |
| Immune response / HMGB1/TLR signaling pathway | 1.253E-02 | 1 | 35 |
|  |  |  |  |
| **S28D vs S35D (negatively affected from S28D to S35D)** |  |  |  |
| Immune response/ IFN alpha/beta signaling pathway | 2.876E-03 | 1 | 24 |
|  |  |  |  |
| **S35D vs21W35D (positively affected from S235D to 21w35D)** |  |  |  |
| Immune response / IFN alpha/beta signaling pathway | 1.300E-05 | 4 | 24 |
| Cell adhesion / ECM remodeling | 2.911E-04 | 4 | 52 |
| Immune response_Antiviral actions of Interferons | 2.911E-04 | 4 | 52 |
| Glutathione metabolism | 6.859E-04 | 4 | 65 |
| Glutathione metabolism / Human version | 7.268E-04 | 4 | 66 |
| Glutathione metabolism / Rodent version | 9.577E-04 | 4 | 71 |
| Immune response / Oncostatin M signaling via MAPK in mouse cells | 1.298E-03 | 3 | 35 |
| Immune response / Oncostatin M signaling via MAPK in human cells | 1.528E-03 | 3 | 37 |
| Cell adhesion / Cell-matrix glycoconjugates | 1.651E-03 | 3 | 38 |
| Development / ERBB-family signaling | 1.781E-03 | 3 | 39 |
|  |  |  |  |
| **S35D vs21W35D (negatively affected from S35D to 21W35D)** |  |  |  |
| Regulation of lipid metabolism / Regulation of acetyl-CoA carboxylase 1 activity | 1.217E-02 | 1 | 17 |
| in lipogenic tissue |  |  |  |
| Regulation of lipid metabolism / Regulation of acetyl-CoA carboxylase 1 activity | 1.217E-02 | 1 | 17 |
| in keratinocytes |  |  |  |
| Development / Delta- and kappa-type opioid receptors signaling via beta-arrestin | 1.643E-02 | 1 | 23 |
| Development / Mu-type opioid receptor signaling via Beta-arrestin | 1.714E-02 | 1 | 24 |
| Development / Angiotensin signaling via beta-Arrestin | 1.785E-02 | 1 | 25 |
| Development / Signaling of Beta-adrenergic receptors via Beta-arrestins | 1.856E-02 | 1 | 26 |
| Regulation of lipid metabolism / RXR-dependent regulation of lipid metabolism | 2.139E-02 | 1 | 30 |
| via PPAR, RAR and VDR |  |  |  |
| Immune response / IL-12-induced IFN-gamma production | 2.562E-02 | 1 | 36 |
| Regulation of lipid metabolism / PPAR regulation of lipid metabolism | 2.983E-02 | 1 | 42 |
| Regulation of metabolism / Role of Adiponectin in regulation of metabolism | 3.053E-02 | 1 | 43 |
|  |  |  |  |
| **21W28D vs21W35D (positively affected form 21W28D to 21W35D)** |  |  |  |
| Immune response / Production and main functions of biologically active | 1.379E-04 | 3 | 50 |
| leukotrienes and Lipoxin A4 |  |  |  |
| Cell cycle / Regulation of G1/S transition (part 2) | 1.276E-03 | 2 | 26 |
| Immune response / CD137 signaling in immune cell | 1.589E-03 | 2 | 29 |
| p53 signaling in Prostate Cancer | 2.056E-03 | 2 | 33 |
| Cell cycle / Regulation of G1/S transition (part 1) | 2.721E-03 | 2 | 38 |
| Development / IGF-1 receptor signaling | 5.044E-03 | 2 | 52 |
| Immune response / CCL2 signaling | 5.430E-03 | 2 | 54 |
| IGF family signaling in colorectal cancer | 6.667E-03 | 2 | 60 |
| Transcription / Role of VDR in regulation of genes involved in osteoporosis | 6.885E-03 | 2 | 61 |
| Cell cycle / Nucleocytoplasmic transport of CDK/Cyclins | 2.856E-02 | 1 | 14 |
|  |  |  |  |
| **21W28D vs21W35D (negatively affected form 21W28D to 21W35D)** |  |  |  |
| Oxidative phosphorylation | 7.333E-04 | 5 | 105 |
| Glutathione metabolism | 9.347E-04 | 4 | 64 |
| Glutathione metabolism / Human version | 9.909E-04 | 4 | 65 |
| Glutathione metabolism / Rodent version | 1.308E-03 | 4 | 70 |
| Transcription / Role of heterochromatin protein 1 (HP1) family in transcriptional | 2.528E-03 | 3 | 40 |
| silencing |  |  |  |
| Proteolysis / Role of Parkin in the Ubiquitin-Proteasomal Pathway | 1.157E-02 | 2 | 24 |
| Translation / Regulation of translation initiation | 1.453E-02 | 2 | 27 |
| Development / Inhibition of angiogenesis by PEDF | 1.891E-02 | 2 | 31 |
| Cytoskeleton remodeling / Keratin filaments | 2.507E-02 | 2 | 36 |
| Influence of low doses of Arsenite on Glucose stimulated Insulin secretion in | 2.507E-02 | 2 | 36 |
| pancreatic cells |  |  |  |
